# Adherence to oral anticancer therapy of oncology patients attending the pharmaceutical office of a public hospital in the lower Amazon region

**DOI:** 10.3332/ecancer.2022.1417

**Published:** 2022-07-01

**Authors:** Yasmim Portela Machado de Aguiar, Adjanny Estela Santos de Souza, Christian Diniz Lima e Silva, Sândrea Ozane do Carmo Queiroz

**Affiliations:** 1 Pará State College/ Center of Biological Sciences and Health, Multiprofessional câncer care residency. Santarém, PA 68040-090, Brazil; 2 Regional do Baixo Amazonas Hospital Dr. Waldemar Pena, Santarém, PA 68040-090, Brazil

**Keywords:** adherence, oral anti-anticancers, cancer

## Abstract

**Introduction:**

Oral anticancer therapy has the benefit of allowing cancer patients to carry out their treatment at home, without being inconvenienced or being at risk of nosocomial infection. However, non-adherence is a public health problem that contributes to the clinical decline of the patient and there are no studies submitted on the adherence of cancer patients to oral anti-anticancer agents in Santarém, PA.

**Objective:**

In view of this, the general objective of this work was to evaluate this oral medication adherence.

**Methods:**

The methodology consisted of a cross-sectional documentary study with a quantitative approach of patients seen at the pharmaceutical office. The Morisky–Green test was used to analyse the degree of adherence and descriptive and inferential statistics were used (*p* < 0.05).

**Results:**

Patients’ adherence to antitumour therapy was not 100%, the majority belonging to females; men were the most adherent, showing adverse reactions to anti-anticancer agents (*p* = 0.0096); comorbidity (*p* = 0.0202) negatively impacted adherence.

**Conclusion:**

It is necessary to adopt new clinical procedures that can contribute to the management of these variables that prevent adherence, in order to improve the effectiveness of the treatment of these patients. There are circumstances that go beyond the aspects inherent to the patient, so it is also relevant to research the external factors that influence the individual’s behaviour, such as the duration of therapy and the consequences of the treatment in the patient’s routine.

## Background

Non-adherence to oral anticancer medication is one of the health problems that impacts society the most, especially in developing countries. According to the World Health Organisation, only 50% of the patients with chronic diseases are adhere to treatment [1–3]. The same occurs with patients with cancer (CA) [4–6]. This problem can influence the decline of the patient’s clinical condition, ineffectiveness in the adherence to oral anticancer medication and, in some cases, can cause the individual to die [7].

Oral anticancer therapy is a treatment modality often used by oncologist physicians [8] due to the benefit of allowing cancer patients to carry out their treatment at home, without having to undergo hospitalisation, having a lower risk of hospital infection and a better quality of life, as it can be involved in daily and family activities [9].

The worldwide cost of cancer is not known, but experts estimate it to be billions of dollars annually. This value tends to increase in the coming years to the detriment of the prevalence of this disease and the costs of treatment [10]. However, adherence to the treatment is defined as an activity dependent on human behaviour; in other words, the patient’s ability to correctly follow the information of a health professional about their pharmacological and non-pharmacological treatment.

In Brazil, the expected occurrence for the development of malignant neoplasms in the years 2020–2022 is approximately 625,000 new cases [11]. Cancer is ranked sixth among the main causes of death in the world [4]. It can be characterised as a set of more than 100 pathologies that consists of malignant, unrestrained and disorganised cell growth, capable of invading tissues and organs [12].

An increasing number of people with malignant neoplasms are put on protocols of oral chemotherapy treatment, which occurs at home. This intensifies how much the patient needs to receive technical guidance on their home therapy [13]. Among the main scales available in the literature (Haynes test, adherence to refills and medications and beliefs about medications) to monitor oral medication adherence, the Morisky–Green test (MGT) is highlighted, as it is the most used instrument in Brazil due to its practicality [14].

However, there is no 100% perfect service in the Unified Health System, but follow-up and monitoring of the adherence are included in this reality. Evidence is lacking for a gold standard method for measuring adherence [4, 15–17]. These are the barriers for those working in the health field, and the instrument methods for assessing adherence are partial because they present advantages and disadvantages (subjectivity) [18].

This questionnaire is used in the pharmaceutical office of the hospital under study, at the moment of pharmaceutical consultation, although the evaluation of its translation in the Portuguese language has not been found in the literature. As it is a tool with subjective questions, a way of minimising this problem was analysed, so it was verified how often the patients went to the pharmaceutical office to receive oral anticancer agents, in order to verify the veracity of the answers attributed during the application of this test.

As the failure in the drug treatment resulting from non-adherence contributes to the progression of this pathology or the loss of a pharmacological alternative, it is important to investigate the clinical variables, as they go beyond the socio-demographic characteristics of the target audience and make it possible to see other factors that may interfere with the treatment. The education of cancer patients is a considerable socio-demographic aspect. The possibility exists that cancer patients with a low level of education have difficulty in understanding the importance of the treatment and have low financial resources to perform health screening or invest in healthy living habits.

Due to cancer presenting a multifactorial etiological characteristic, it becomes necessary to identify the main neoplasms that affect Brazilians in Western Pará, in order to work on preventive measures in the future. Therefore, it is observed that the lack of adherence can also result in the waste of public money given to this region for oral chemotherapy, as patients fail to carry out the treatment they are supposed to. In addition, there is a lack of studies regarding the population’s adherence. So, it is relevant to analyse the adherence to this therapy.

Given these hypotheses, the general objective of the research was to evaluate the oral medication adherence of cancer patients treated at the Regional Hospital of Baixo Amazonas Dr. Waldemar Penna (HRBA). The aim was also to define the profile of cancer patients who received oral anticancer drugs at the HRBA pharmaceutical office, using the following socio-demographic and clinical variables: ethnicity, sex, age, education level, level of adherence, oncological pathology, comorbidity, oral anticancer medication, adverse reactions to medications (ADR) and intentional forgetfulness of the patient regarding taking the medications. The variables also included the search for an association between adherence rate with age, sex, education and comorbidity and to investigate whether the adverse reactions of oral anticancer agents interfere with the level of adherence.

The HRBA is a reference in the Northern region for the treatment of malignant tumours, being the first public hospital in this region of Brazil to obtain a certificate of maximum quality, accredited with excellence by the National Accreditation Organisation [13]. Santarém is the third largest city in the State of Pará. It is a metropolitan region according to the Brazilian Institute of Geography and Statistics (Instituto Braasileiro de Geografia e Estatística-IBGE). The gross domestic product of this city is 3 billion and 788 million reais. Approximately 50% of this value is related to the tertiary sector, which consists of services and commerce. Industry results in 15% and extractivism in 35%. The city’s development conflicts a fundamental factor, i.e., the infrastructure needs to be improved. Deficiencies in water and energy supply are still precarious and make it difficult to install industries in the city. There are more than 700 km of road and only 36% are paved. Many companies are reluctant to come to Santarém because of electricity, which is the main input for an industry [19].

## Methods

This study is a cross-sectional documentary study with a quantitative approach, conducted in the pharmaceutical office, with cancer patients treated at this location and who received oral anticancer agents during June–September 2019. The population studied composed of 92 patients with cancer, while the size of the sample consisted of the inclusion of 69 patients because 23 were excluded.

The study included individuals from 18 years of age who underwent treatment with oral anticancer agents. The 23 patients excluded were those who progressed to death, who were not in a position to establish communication and who had asked other people to seek medication in the office, such as companions or representatives of support houses.

To obtain the data, socio-demographic and clinical information were collected from the medical records of these patients. The degree of adherence to anticancer oral medication was estimated through the MGT applied in the pharmacist’s office at the time of dispensing these drugs. MGT consists of a questionnaire with four questions, to which the respondents could answer in a dichotomous way (yes/no) [14]. If, in this evaluation, there were questions with only negative answers, the patients were categorised as adherent and as non-adherent if there was at least one positive answer [12, 15].

Regarding data analysis, they were organised in spread sheets in Microsoft Excel 2016 and evaluated through the BioEstat 5.0 software. Descriptive statistics were used for organisation and tabulation of data through tables and figures with absolute and percentage values, as well as inferential statistics, which demanded the application of the normality test first.

Due to the heterogeneous distribution of the data, the chi-square (QQ) and Fisher’s exact tests were chosen to verify whether the patients’ socio-demographic and clinical characteristics influenced adherence to drug treatment, considering *p* < 0.05. In view of this, the null hypothesis of the study is that the adherence of the patients to therapy does not depend on the variables mentioned and in the alternative hypothesis the rate of pharmacological adherence depends on these factors.

The study was approved by the research ethics committee of the State University of Pará, campus Santarém, according to opinion 3,835,038 and CAAE 26543819.6.0000.5168.

## Results

After defining the profile of the studied population and analysing their medical records, it was identified that the majority of the studied patients were female (85.51%), yellow ethnicity (82.61%), aged less than 60 years (71.01%) and had high school or higher education (59.42%) (Table 1).

The most frequent cancer pathology consisted of breast cancer (78.26%), followed by prostate cancer (8.70%) and chronic myeloid leukaemia (CML) (4.35%). Anastrozole (40.58%) and Tamoxifen citrate (39.13%) were the most commonly used oral anticancer therapy (39.13%) (Table 2).

Table 3 shows that 53.6% of the participants had adequate adherence to anticancer treatment because they answered no to all questions in the questionnaire. The main causes of non-adherence to pharmacological treatment consisted of carelessness regarding the correct time to take the medication and forgetfulness, both with 42%.

According to Table 4, men had a higher prevalence of adherence to pharmacological treatment (60%), were aged 60 years and above (65%), had an education up to elementary school (57.14%) and were without comorbidities (69.70%). Clinical studies suggest that it cannot be said that sex, age and education are variables that influence adherence to the treatment of these patients (*p* > 0.05), and most of them had unintended forgetfulness.

However, there was a significant association between adherence and absence of comorbidities (*p* = 0.0202). It was also detected that patients who had less than three adverse reactions to anticancer agents had better adherence to treatment, with a statistically significant difference (*p* = 0.0096). Thus, the results suggest that the lower number of ADRs and the absence of comorbidities impact pharmacological persistence.

So, these results indicate that ADR and comorbidity influence adherence to oral anticancer therapy (*p* <0.05). Individuals who had a lower number of ADRs were adherent and the majority of those who were non-adherent had comorbidities.

Although ADRs had an impact on the effectiveness of therapy, there was no significant association between the drugs used in the treatment of adherent and non-adherent groups (Table 5).

## Discussion

Oral anticancer therapy is still poorly studied in Brazil, as more Brazilian studies on this topic are lacking. In this research, we evaluated that the adherence of cancer patients to oral anticancer treatment at the HRBA pharmaceutical office is not 100%, despite 53.6% of the 69 participants with cancer being adherent. It is suggested that the fact that most patients are adherent demonstrates that the severity of the disease represents a major force that positively influences adherence to oral anticancer medication. However, good patient education with oral medication and a good relationship with the pharmacist or family members may also have encouraged adherence.

As for the quality of the MGT test used by the researchers, they found that it has been a tool for monitoring adherence applied for many years, validated in the United States to assess the adherence of patients undergoing treatment with antihypertensive drugs [20]. Cancer patients have similar characteristics to patients who are hypertensive in terms of pathological chronicity, past clinical history and prolonged progress, asymptomatology, clinical manifestations with recurrences, criticality and progression to disability or death ^7^.

Through the MGT, obstacles to adherence were evidenced, which consisted of the time to take the medication (42%), as the patients forgot to take medication, and others stopped taking the medication because they felt better or stopped the treatment on their own initiative, after feeling well (4.3%).

Regarding the socio-demographic results of the adherent group, men (60%) with prostate cancer and older than 60 years are adherent to anticancer hormone therapy and the highest frequencies of the non-adherent population are women (47.46%) with breast cancer on hormone therapy and less than 60 years old, with the majority of the population being mixed race.

The results of a study with cancer patients (*n* = 222) showed that 89% of the adherent group were women with the same pharmacological approach, aged less than or equal to 60 years [20] and the non-adherent population consisted of 23% males and 11% females. In another study (*n* = 225), women (86.4%) also had better adherence to oral anticancer therapy compared to men (68.9%) [21]. As found in these studies, we perceive a contradiction with the present research. This may be related to the ADRs that women may have with hormonal therapy, when compared to the ADRs of male treatment.

In view of this, it was ensured that non-adherence to antitumour agents is not exclusive to the population studied. Evidence from studies on the adherence to anti-anticancer agents through this same route of administration shows that cancer patients from other countries and regions of Brazil are also negligent with oral therapy, because they stopped taking their medications at some point during treatment [7, 15, 20–23] and the same occurred with the participants in this work.

The assessment of adherence to pharmacological therapy with oral anticancer agents has been discussed for some years by researchers, who carried out an analysis with various types of oncological pathologies [7, 21, 24, 25], and the rate of this adherence, as in this study, has not fully reached a 100% adhesion, obtaining results below 20% [7, 21, 25, 26].

Despite the ethnicity in this research, it diverges with information obtained by researchers who investigated 197 women on this issue, and demonstrated that Caucasian women with breast cancer had a longer survival time when compared to black women affected by this same cancer. This shows the biological characteristics of the malignant tumour and low adherence to hormonal therapy [27]. However, in the study, the sample size was smaller and with few black patients, a fact that may justify this result.

Evidence from a randomised study on schooling (*n* = 48) showed a statistically significant relationship between literacy and adherence to anticancer agents; in other words, this variable impacted on the quality of adherence to treatment, literate individuals were more assiduous with self-administered therapy [28]. However, this variable did not affect the adherence behaviour of the investigated patients and this research had another methodological approach.

However, it was noted that the results found in a systematic review show disagreement about education, age, sex and ethnicity, because some studies suggest that relevant factors and others are unfavourable to adherence [29]. In this sense, the Brazilian literature has found that these factors may also not influence adherence [7, 30]. Although females are the most affected by cancer in the region of Baixo Amazonas, men are more adherent to hormone therapy.

Another similarity of this research was identified in what is related to the main types of cancers and anticancer therapy in use. The most discussed data were from women with breast cancer treated with oral hormone therapy [23, 31, 32], patients with CML [33] using Imatinib mesylate and men with prostate cancer on oral hormone therapy [21]. Coincidentally, these pathologies were the main ones observed in the population studied and it was noted that these malignant tumours are among the cancers that most affect Brazilians and Americans.

With regard to the unintentional behaviour of forgetting the treatment found in that study, it is interesting. Although some patients stated that they stopped taking the medication because they felt better, most reported not having the intention to forget about taking the drugs and this may be related to the way in which the patient deals with the disease and waiting for their pharmacological results, because cancer is often interpreted as an imminent risk of death that triggers fear. In view of this, the psychological factors of the participants must be investigated by the pharmacist in order to be discussed by the multiprofessional health team, mainly with psychologists, in order to avoid new barriers that may hinder oral anticancer therapy.

Still on these discussions, a study related to the behaviour of the use of this therapy showed a result similar to this article due to the higher frequency (58.9%) of non-adherents also not having intentional forgetfulness and 33.9% reported remembering the time but not taking the medicine for another reason [34]. Research report that psychosocial variables are important factors that influence the clinic. Cancer patients with negative thoughts, poor communication and poor quality of life are prone to non-adherence of treatment [35].

The occurrence of ADR and comorbidities were the most relevant clinical variables that contributed to non-adherence. These physiological characteristics are similar to other bibliographic collections on therapy with oral anticancer agents [5, 36, 37]. The most reported ADRs were hot flushes, headache, myalgia and vaginal dryness.

Brazilian studies have found hot flushes or heat as the most frequent ADR that negatively influences the treatment [37, 38]. Others related the perception of adherence to anticancer therapy as unsatisfactory after discovering that ADRs are one of the clinical factors that potentially affect pharmacological adherence [22, 23] and are one of the causes of treatment withdrawal [28]. Therefore, this fact corroborates the findings of this article; for this reason, measures of the clinical management against these unwanted effects of therapy should be discussed by the pharmacist in partnership with the health professionals involved in the process of care for cancer patients.

The curious thing about this is that non-adherent women are in the age group less than 60 years, a phase of adult life that is probably more sexually active, and perhaps for this reason they neglect hormone therapy due to ADRs such as hot flushes and vaginal dryness caused by Anastrozole or Tamoxifen citrate. However, we were unable to relate the interference of these drugs with adherence, but these unwanted effects were reported by some women during treatment.

In reference to comorbidity, it was shown that it can influence oral medication. The situation that made the authors accept the hypothesis was that if the patient has more than one responsibility about being assiduous in therapy for other diseases, during this process they may neglect treatment against cancer. So pharmaceutical care focused on non-pharmacological measures, how to guide these patients on the importance of oral anticancer therapy, coexisting diseases and the consequences of non-adherence to treatment for both pathologies are essential. The prevalence of malignant tumours interconnected with other chronic diseases implies decision-making about treatment and its outcome [39], due to the comorbidity accelerating the development of the tumour and disrupting the results of the therapy against cancer [5].

There is a negative impact on the health of the individual who has more than one clinical condition during treatment with anticancer agents. The prevalence of being an oncological patient with other pathologies varies from 0.4% to 90% and the profile is older individuals, which is understandable, because as the illnesses appear the cancer therapy becomes more difficult [40]. Once again, results compatible with this work are identified, because in the studied population there are patients aged 60 years or older and undergoing antitumour treatment, which may be compromised to the detriment of non-adherence.

In the United States, it was possible to draw a profile of cancer patients with comorbidities. Those with breast and prostate cancers had other underlying diseases and this prevalence of comorbidity was similar to the population average (30%–32% of those aged 66 years and above). This fact contributes to the pharmacotherapeutic decline and the individual’s survival [41, 42].

In view of the above, the data that contributed to the lack of adherence in this work in the face of pharmacological treatment are considered negative, as the lack of commitment to self-administered chemotherapy affects the poor prognosis of the patient and it is highlighted how much it is a priority to monitor treatment adherence.

In this sense, there are situations that, although not explored, should be taken into account when the problem is not adhering to the treatment, such as ethical, socioeconomic, cultural and religious aspects, the fear of dealing with cancer and even the type of relationship between the patient and health professional all influence medication adherence [18, 43].

Based on this assumption, the financial problems faced by cancer patients must also be analysed, since in addition to requiring a specific treatment location, such as a high and medium complexity hospital, many of them do not reside in Santarém, PA, need to be away from work, leave their homes in search of treatment, pay for other expenses during the journey to the hospital and sometimes do not have funds for temporary housing, needing to stay in support homes until they seek monthly medication at the pharmacist’s office.

Incidentally, the hormone therapy (Tamoxifen and Anastrozole) that was the most used presents as a disadvantage due to the prolonged treatment time, which can go up to 5 years, and due to this many patients are unable to maintain a good attendance over time and end up interrupting the therapy because they believe it is no longer needed. This fact makes the individual the main person responsible for themselves [44]. As a result and to avoid this behaviour, the pharmacist has the potential to educate patients to be persistent with the treatment and avoid the loss of a pharmacological alternative that could possibly be the only option.

This set of factors can generate stress in the patient, due to the excess of worries and perhaps uncertainties about the health/disease process, which may unconsciously influence adherence. Thus, as the pharmacists dispense anticancer agents, they need to have a holistic view of the patient, be empathetic, identify the patient’s clinical needs and demonstrate security, since the patient has the right to choose and their decisions need to be respected.

Regarding the cost of oral cytostatics, it is known that billions of dollars are estimated ^10^, and in the hospital studied, this investment amounted to R$2,233,590,41 in 2020. This value is related to the public money invested in this therapeutic approach. For this reason, supervision of adherence to oral treatment is emphasised, with the intention of avoiding the waste of this public budget, which is very useful for the region of Western Pará.

It is inferred that, from now on, it will be possible to implement resolutions to improve the medication adherence of the non-adherent population to cancer treatment. The study will allow health professionals who care for these patients a differentiated clinical perception, in order to awaken triggers in these professionals when they attend patients who report having ADR and comorbidities, for the purpose of early identification of the factors that tend towards therapeutic non-adherence.

When this perception does not occur and the patient has a poor adherence, the doctor may think that the failure in therapy is due to the progression of the disease and ineffectiveness of the anticancer medication and thus implement a new pharmacotherapy, resulting in the loss of a treatment that could be used [6].

## Conclusion

Despite the results of oral medication adherence of cancer patients being relatively high, it has been found that 46% of these individuals do not adhere to oral anticancer therapy; this reality is a problem. Therefore, the alternative hypothesis of comorbidity and ADR negatively influencing therapy is accepted. This again intensifies the need to consider the use of therapeutic goals to manage these impediments to adherence, mainly by the pharmacist who is the technician in charge of dispensing these medications.

When generating the profile of this population, it was observed that public policies must be implemented to work on the promotion and prevention of cancer, mainly in the health screening of breast, prostate and CML cancers, which are the most prevalent in the lower Amazon region.

The obstacles to adherence to self-administered chemotherapy prompted several questions for future work because there are circumstances that go beyond the aspects inherent to the patient, and it is necessary to research the external factors that influence the individual’s behaviour, such as the duration of the therapy and the consequences of the treatment on the patient’s routine. Social and financial variables also need to be studied, as they can be barriers that hinder oral anticancer therapy, such as proposing, for example, the bimonthly dispensation of endocrine therapy instead of semi-annually for those women who do not reside in Santarém, PA, in order to avoid interruption of therapy.

Finally, the limitations of the work are due to the lack of studies in the national and international research indexes on the analysis of adherence to oral anticancer therapy in cancer patients. Also including the small number of male compared to female participants and the risk that patients who forgot to take anticancer drugs are, for example, embarrassed or afraid to admit this fact to the pharmacist.

## Conflicts of interest and funding

This work has no conflicts of interest or funding.

## Figures and Tables

**Table 1.** Socio-demographic variables of the patients undergoing oral anticancer therapy.

Socio-demographic variables	Total		Male		Female	
	*N*	%	*n*	%	*n*	%
**Sex**	69	100.00	10	14.49	59	85.51
**Ethnicity**						
White	8	11.59	2	2.90	6	8.70
Indigenous	1	1.45	0	0.00	1	1.45
Black	3	4.35	2	2.90	1	1.45
Yellow	57	82.61	6	8.70	51	73.91
**Age**						
Under 60 years old	49	71.01	3	4.35	46	66.67
Greater than or equal to 60 years old	20	28.99	7	10.14	13	18.84
**Education**						
Up to primary	28	40.58	5	7.25	23	33.33
High or higher	41	59.42	5	7.25	36	52.17

Source: Research data, 2021

**Table 2.** Medical diagnosis and pharmacotherapy used by cancer patients.

Variables		*N*	%
**Oncological pathology**			
	Breast cancer	54	78.26
	Prostate cancer	6	8.70
	Chronic myeloid leukaemia	3	4.35
	Desmoid cancer	1	1.45
	Gastrointestinal cancer	1	1.45
	Multiple myeloma	1	1.45
	Rectum cancer	1	1.45
	Kidney cancer	1	1.45
	Thyroid cancer	1	1.45
**Oral anti-anticancer medication**			
	Anastrozole	28	40.58
	Tamoxifen citrate	27	39.13
	Imatinib mesylate	4	5.80
	Bicalutamide	4	5.80
	Abiraterone	2	2.90
	Thalidomide	1	1.45
	Sorafenib	1	1.45
	Pazopanib	1	1.45
	Capecitabine	1	1.45

Source: Research data, 2021

**Table 3.** Classification of adherence by the MGT.

Morisky–Green test	Yes		No	
	*N*	%	*N*	%
1 - Have you ever forgotten to take the medicine for your disease?	29	42.0	40	58.0
2 - Have you ever forgotten the time of taking your medication?	29	42.0	40	58.0
3 - Have you stopped taking the medicine for your disease because you feel better?	3	4.3	66	95,7
4 - Have you ever stopped taking the medicine for your disease, on your own initiative, after feeling better?	3	4.3	66	95.7
**Adherence rating**	***N***		**%**	
Adherent	37		53.6	
Non-adherent	32		46.4	

Source: Research data, 2021

**Table 4.** Factors that can influence adherence to oral anticancer therapy.

Variables	Total		Adherent		Non-adherent		P
	*N*	%	*N*	%	*N*	%	
**Sex**							
Male	10	100.00	6	60.00	4	40.00	0.742**
Female	59	100.00	31	52.54	28	47.46	
**Age**							
Under 60 years old	49	100.00	24	48.98	25	51.02	0.226*
Greater than or equal to 60 years old	20	100.00	13	65.00	7	35.00%	
**Education**							
Up to primary	28	100.00	16	57.14	12	42.86	0.628*
High or higher	41	100.00	21	51.22	20	48.78	
**No. of adverse reactions**							
≥3	9	100.00	1	11.11	8	88.89	0.0096**
<3	60	100.00	36	60.00	24	40.00	
**Intentional forgetfulness**							
Yes	3	100.00	-	-	3	100.00	-
No	29	100.00	-	-	29	100.00%	-
**Comorbidities**							
Yes	36	100.00	14	38.89	22	61.11	0.0202*
No	33	100.00	23	69.70	10	30.30	

* QQ test;

** Fisher’s exact test

Source: Research data, 2021

**Table 5.** Association of adherence with the oral anti-anticancer used.

Anti-anticancer Medication Oral	Adherent		Non-Adherent		
	*N*	%	*N*	%	*p*
TOTAL	37	53.6	32	46.4	
					0.774*
Anastrozole	15	53.6	13	46.4	
Tamoxifen citrate	14	51.9	13	48.1	
Imatinib mesylate	3	75.0	1	25.0	
Bicalutamide	2	50.0	2	50.0	
Abiraterone	1	50.0	1	50.0	
Thalidomide	0	0.0	1	100.0	
Sorafenib	0	0.0	1	100.0	
Pazopanib	1	100.0	0	0.0	
Capecitabine	1	100.0	0	0.0	

* QQ test

Source: Research data, 2021
